# Additional Attachment of the Semitendinosus and Gracilis Muscles to the Crural Fascia: A Review and Case Illustration

**DOI:** 10.7759/cureus.3116

**Published:** 2018-08-07

**Authors:** Asad Rizvi, Joe Iwanaga, Rod J Oskouian, Marios Loukas, R. Shane Tubbs

**Affiliations:** 1 Medicine, St. Georges University School of Medicine, St. Georges, GRD; 2 Medical Education and Simulation, Seattle Science Foundation, Seattle, USA; 3 Neurosurgery, Swedish Neuroscience Institute, Seattle, USA; 4 Anatomical Sciences, St. George's University, St. George's, GRD; 5 Neurosurgery, Seattle Science Foundation, Seattle, USA

**Keywords:** semitendinosus, gracilis, acl reconstruction, crural fascia, deep fascia, accessory bands

## Abstract

The semitendinosus and gracilis muscles insert primarily onto the superior medial aspect of the tibia. These tendons can be harvested for anterior cruciate ligament reconstruction, and knowledge of their accessory attachments is important for the success of such harvesting procedures. Here, we present a case illustration and review of the attachment of these muscles into the crural fascia (deep fascia of the leg), which is often an underappreciated insertion site.

## Introduction

The semitendinosus is one of the hamstring muscles located in the posterior-medial thigh. It originates from the inferior-medial impression on the superior part of the ischial tuberosity and shares a tendon with the biceps femoris. The long distal tendon of the semitendinosus starts below the mid-thigh on the posterior of the semimembranosus and turns around the medial condyle of the tibia to insert onto the superomedial aspect of the tibia. The tendons of the semitendinosus, sartorius, and gracilis eventually conjoin to form the pes anserinus. The semitendinosus can also attach to the crural fascia of the leg and is usually the only attachment to this fascia [1].

The gracilis muscle arises from the pubic bone and descends along the medial thigh. This medial compartment muscle travels distally and, as mentioned above, unites with the semitendinosus and sartorius to form the pes anserinus, which attaches to the medial tibia.

The tendons of the semitendinosus and gracilis muscles can be harvested for anterior cruciate ligament reconstruction, and awareness of the various accessory attachments of their distal tendons is important during such harvesting procedures [2]. Herein, we present a case illustration, review the often-underappreciated additional attachment of the semitendinosus and gracilis muscles to the crural fascia, and discuss other variations reported in the literature.

## Case presentation

**Figure 1:**
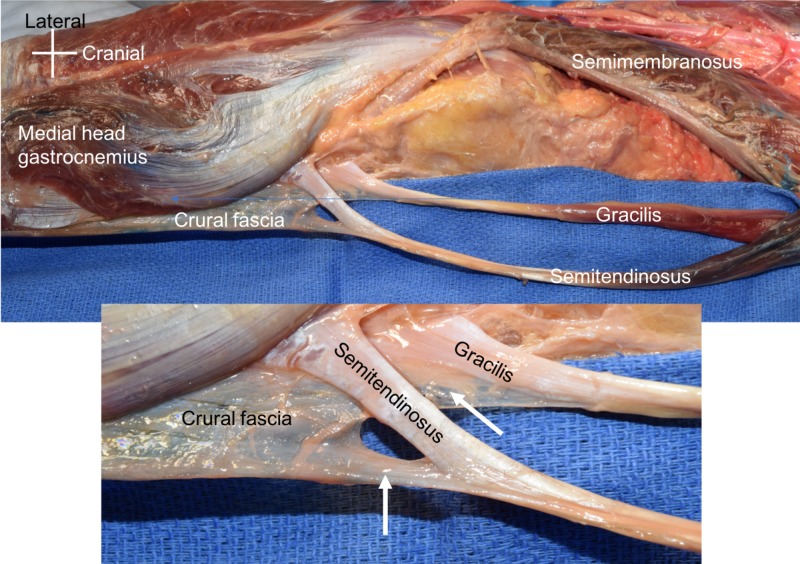
Cadaveric specimen reported herein Note the semitendinosus and gracilis distal tendons inserting not only into their normal site but also into the crural fascia (arrows in the zoomed in view).

During a routine dissection of the left medial thigh, leg, and knee of a cadaver, an unusual additional attachment of the semitendinosus and gracilis muscles was found into the crural fascia (Figure 1). The cadaveric specimen was of a female who had died at the age of 72 years. The muscles’ proximal attachment and course were normal as was their distal attachment onto the tibia via the pes anserine. However, the distal attachment also included a variant attachment into the crural fascia of the leg. The nerve innervation to the semitendinosus was from a tibial branch derived from the sciatic nerve and to the gracilis from the obturator nerve. No other anatomical variations were identified in this cadaveric specimen.

## Discussion

As mentioned above, tendons such as from the semitendinosus and gracilis can be harvested for anterior cruciate ligament reconstruction. This procedure is usually performed through a small incision and involves the use of a tendon stripper. Variations in the attachment of the semitendinosus and gracilis muscle through accessory bands to, for example, the crural fascia can cause some difficulty in harvesting the tendon, and knowledge of the location of these attachments can help in avoiding potential complications, e.g., premature tendon amputation during the procedure [2].

An accessory tendon similar to the case presented here has been reported to arise before the semitendinosus tendon and to fuse with the gracilis tendon and was found to attach to the crural fascia [3]. A fascial sling that invests the semitendinosus has also been described [4]. Several other accessory bands arising from the semitendinosus muscle and displaying high variability have also been reported in the literature, including a large band occurring proximal to the insertion of the semitendinosus tendon on the tibia and attached to the fascia overlying the gastrocnemius, i.e., the crural fascia [2,5]. Other smaller bands can arise approximately 10 cm proximal to the insertion onto the tibia and insert into the popliteal fascia, gastrocnemius, or gracilis tendon [2].

Other variations in the anatomy of the semitendinosus include a separation of the semitendinosus from the biceps femoris; partial fusion of the semitendinosus and the semimembranosus; and accessory bands from the biceps femoris, sacrotuberous ligament, ischial tuberosity, or coccyx attaching to the semitendinosus muscle. Additionally, an accessory muscular slip called the tensor fasciae suralis arises from the belly of the semitendinosus and ends in a tendon connecting to the crural fascia [6-10]. The gracilis might also have additional fibers connected to the fascia lata.

## Conclusions

An awareness of the accessory attachments (e.g. crural fascia) of the semitendinosus and gracilis muscles is important for the success of the harvesting procedure. Here, we highlight a case illustration so that clinicians are more cognizant of the possibility of this underreported attachment.
